# c-Jun as a one-way valve at the naive to primed interface

**DOI:** 10.1186/s13578-023-01141-0

**Published:** 2023-10-14

**Authors:** Dongwei Li, Ling Luo, Lin Guo, Chuman Wu, Ran Zhang, Yuling Peng, Menghua Wu, Junqi Kuang, Yan Li, Yudan Zhang, Jun Xie, Wenxiu Xie, Rui Mao, Gang Ma, Xiuling Fu, Jiekai Chen, Andrew P. Hutchins, Duanqing Pei

**Affiliations:** 1 Key Laboratory of Biological Targeting Diagnosis, Therapy and Rehabilitation of Guangdong Higher Education Institutes, The Fifth Affiliated Hospital of Guangzhou Medical University, 190 Kaiyuan Dadao, Huangpu District, Guangzhou, 510799 China; 2 Guangdong Cardiovascular Institute, Guangdong Provincial People’s Hospital, (Guangdong Academy of Medical Sciences), Southern Medical University, Guangzhou, 510100 Guangdong China; 3 Guangdong Provincial Key Laboratory of Stem Cell and Regenerative Medicine, Center for Cell Lineage and Development, Guangzhou Institutes of Biomedicine and Health, Chinese Academy of Sciences, Guangzhou, 510530 China; 4 Bioland Laboratory (Guangzhou Regenerative Medicine and Health Guangdong Laboratory), Guangzhou, 510005 China; 5 Department of Systems Biology, School of Life Sciences, Southern University of Science and Technology, Shenzhen, 518055 China; 6 Laboratory of Cell Fate Control, School of Life Sciences, Westlake University, Yunqi Town, No.18 Longshan Street, Xihu District, Hangzhou, 310024 China

**Keywords:** EpiSCs, c-Jun, Naïve to primed transition, Primed to naïve transition

## Abstract

**Background:**

c-Jun is a proto-oncogene functioning as a transcription factor to activate gene expression under many physiological and pathological conditions, particularly in somatic cells. However, its role in early embryonic development remains unknown.

**Results:**

Here, we show that c-Jun acts as a one-way valve to preserve the primed state and impair reversion to the naïve state. c-Jun is induced during the naive to primed transition, and it works to stabilize the chromatin structure and inhibit the reverse transition. Loss of c-Jun has surprisingly little effect on the naïve to primed transition, and no phenotypic effect on primed cells, however, in primed cells the loss of c-Jun leads to a failure to correctly close naïve-specific enhancers. When the primed cells are induced to reprogram to a naïve state, these enhancers are more rapidly activated when c-Jun is lost or impaired, and the conversion is more efficient.

**Conclusions:**

The results of this study indicate that c-Jun can function as a chromatin stabilizer in primed EpiSCs, to maintain the epigenetic cell type state and act as a one-way valve for cell fate conversions.

**Supplementary Information:**

The online version contains supplementary material available at 10.1186/s13578-023-01141-0.

## Background

c-Jun is a member of a family of transcription factors (TFs) known as activator protein-1 (AP-1) factors implicated in multiple cellular processes, including cell cycle, apoptosis and cell proliferation [1, 2]. c-Jun plays a critical role in early embryonic development as c-Jun-/- embryos die at the mid- to late-gestation stage [3]. A role for c-Jun earlier in development has not been established, although it is expressed post-implantation. Preimplantation embryos and mouse embryonic stem cells (ESCs) do not express appreciable levels of c-Jun [4], however, treatment of human embryonic stem cells (hESCs), which may represent a slightly later developmental stage, with a c-Jun N-terminal kinase inhibitor (JNKi) promotes differentiation to a definitive endoderm fate [5]. These suggest that c-Jun has complex, perhaps opposing, roles in pre-implantation development.

AP-1/c-Jun is enriched in multi-loop activation hubs, which are involved in regulating cell type-specific genes [6]. Studies involving somatic cell reprogramming have shown that the AP-1/c-Jun motif is enriched in open chromatin that rapidly closes in the early stages of reprogramming [7–9]. These chromatin accessibility dynamics can be disrupted by exogenously expressed c-Jun [9], suggesting that c-Jun is regulating chromatin dynamics. Indeed, c-Jun has been shown to bind open chromatin and maintain chromatin accessibility [10]. In fibroblasts, AP-1/c-Jun and cell-type-specific TFs can recruit BAF (mammalian SWI/SNF) complex to remodel nucleosomes and open chromatin to activate silenced enhancers [11]. Conversely, c-Jun can also recruit nucleosome remodeling and histone deacetylation (NuRD) repressor complex by interacting with Mbd3 to silence AP-1 target genes [12]. Hence c-Jun, like a lot of transcription factors, has both activatory and repressive potential that is potentially involved in cell fate decisions.

**Fig. 1:**
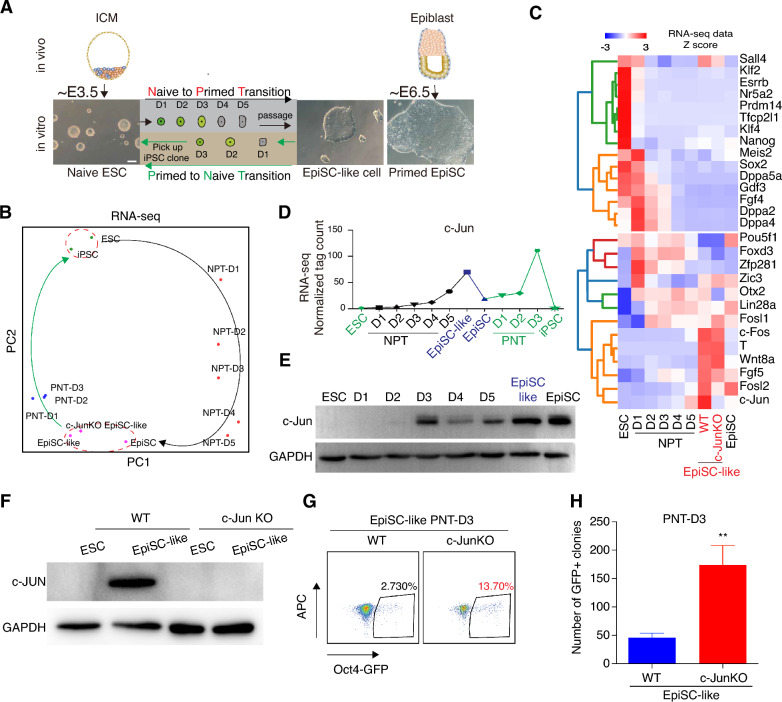
c-Jun inhibits primed to naïve transition. **A**. Schematic for the derive mouse naive ESC, primed EpiSC and induce naïve ESC differentiation into EpiSC-like cells (naive to primed transition, NPT) under EpiSC culture medium (see methods). And induce primed to naive transition (PNT) (see methods). D indicates the day of the naive to primed transition or primed to naive transition. **B**. Principal Component Analysis (PCA) of the gene expression (RNA-seq) during naive to primed transition (NPT) and primed to naive transition (PNT). **C**. Heatmap of naive and primed marker genes expressed during NPT. **D**. RNA-seq data show the c-Jun expression levels in ESC, a typical naive to primed transition and primed to naive transition time course, EpiSC-like cells, EpiSC and iPSCs. D = day. **E**. Western blot of the levels of c-Jun protein in ESC, a typical naive to primed transition time course, and EpiSC. D = day. **F**. Western blot of c-Jun protein in WT ESC, EpiSC-like, c-JunKO ESC and c-JunKO EpiSC-like cells. **G**. Flow cytometry analysis of the Oct4-GFP (OG2) reporter at primed to naïve transition (PNT) day 3. D = day. Scale bar = 100 μm. **H**. Effect of knock out of c-Jun in day 3 primed to naive transition. The number of GFP positive colonies was counted on day 3. D = day. Data from 6 biological replicates from 2 independent experiments and shown as the mean ± SEM. *** indicates P value < 0.001, t-test analysis between the c-Jun knock out EpiSClike and WT EpiSC-like cells.

Mouse pre- and post-implantation embryos have been captured as in vitro models in the form of naive (ESCs), cultured in 2i medium (CHIR and PD0325901) [13], and primed epiblast stem cells (EpiSCs), that are cultured with Activin A and bFGF [14–16]. These two in vitro models serve as a model system to study the molecular mechanisms regulating the transition between E3.5 and E5.5 embryos (Fig. 1A) [17]. Both ESCs and EpiSCs are considered pluripotent, i.e., capable of generating cells of the three germ layers through in vitro differentiation and in vivo teratoma assays, yet they behave quite differently in their capacity to form chimeras and contribute to the germline: naïve ESCs are capable, but EpiSCs have great difficulty in contributing to chimeras and do not contribute to the germline [18, 19]. Therefore, the distinction between naive and primed states are critical to our understanding of early mouse embryo development in general and perhaps cell fate specification in particular.

Importantly, the naïve and primed states function as a powerful model of cell type-conversion as they can be interconverted in vitro relatively easily, in a process called the naïve-to-primed transition (NPT), or the reverse process PNT [16, 20–26]. We have previously shown that c-Jun is not expressed in the naive state, but in MEFs c-Jun acts as a gatekeeper of the somatic fate [4]. However, as primed EpiSCs are still in the process of development and retain potent developmental capacity, it is not clear if c-Jun has a role in the primed state.

In this report, we show that c-Jun regulates the transition between naïve and primed states. Using the interconversions between ESCs and EpiSCs as a model system, we show that they have distinct chromatin accessibility states, and c-Jun plays a unique role in specifying the nuclear architecture of EpiSCs. In EpiSCs, c-Jun helps inhibit closed naïve enhancers and maintains them in a repressed state. Loss of c-Jun leads to incomplete suppression of naïve enhancers which potentiates the cells for more efficient conversion from primed to naïve states. Our studies reveal an unexpected role of c-Jun in the transition between naive to primed states, suggesting that it serves to endow somatic properties in exiting naive cells by establishing and stabilizing chromatin architecture.

## Results

### Induction of c-Jun during the naïve to primed transition

To understand how the naïve state transitions to the primed one, we cultured mouse ESCs in naïve conditions containing LIF and two inhibitors (2i) CHIR99021 (WNT signal pathway inhibitor) and PD0325901 (ERK inhibitor) [13]. Naïve ESCs were differentiated to primed EpiSCs (converted cells are referred to as EpiSC-like cells) by using a protocol involving basal medium containing four small molecules: Activin A, bFGF, XAV939 and IWR [27, 28]. Morphologically, the embryo-derived (EpiSCs) or induced EpiSC-like cells are nearly identical (Fig. 1A, right panels and 1B). Principal component analysis (PCA) on time-course RNA-seq data during the naïve to primed transition (NPT), showed a clear trajectory from ESCs to EpiSCs (Fig. 1B). Naïve-specific pluripotent genes, such as Nanog, Esrrb, Dppa5a were rapidly downregulated, conversely, the primed-specific marker genes Lin28a, Fgf5, T were activated only in the late stage of NPT (Fig. 1C). Interestingly, at both the RNA and protein level, we show that c-Jun was expressed as early as D2 (Day) during NPT, and highly expressed in EpiSC-like cells (Fig. 1D, E).

To study the role of c-Jun in the NPT, we designed two sgRNAs that target two flanking regions of the single exon of c-Jun (Additional file 1: Fig. S1A). We transfected those two sgRNAs into ESCs to knock out c-Jun. As c-Jun is not expressed in ESCs, we converted them to the primed state and confirmed the knockout by Western blot (Fig. 1F). We next looked at RNA-seq of the c-JunKO EpiSC-like cells, and found that the transcriptomes were nearly identical: EpiSC-like versus EpiSCs, R = 0.959; and c-JunKO EpiSC-like versus EpiSCs, R = 0.947 (Additional file 1: Fig. S1B, C). As expected, c-JunKO EpiSC-like cells express normal levels of both pluripotent (Pou5f1, Nanog) and EpiSC-specific (Fgf4) genes that remained expressed at similar levels when c-Jun was knocked out (Additional file 1: Fig. S1B). c-JunKO EpiSC-like cells also clustered by PCA closely with normal EpiSCs and EpiSC-like cells (Fig. 1B). In the ATAC-seq data, chromatin accessibility of c-JunKO EpiSC-like cells was similar with WT EpiSC-like cells and EpiSC (Additional file 1: Fig. S1D). Together these results indicate that c-Jun is not required for NPT nor primed state despite of being unregulated robustly.

### c-Jun knockout promotes primed to naive transition

Whilst c-Jun is not required for the NPT, we next explored if c-Jun is needed for the reverse, primed to naïve transition (PNT). To explore this, we exploited the ability of exogenously expressed Klf4 to drive the PNT and established a Klf4-transgenic mEpiSC line (kEpiSCs), that bears two transgenes: An Oct4-GFP reporter system where GFP is only expressed only in the naïve state [29], and *Klf4* under the control of a CAG promoter that converts EpiSCs to ESCs [23] (Additional file 1: Fig. S2A). When Klf4 was expressed, however, kEpiSCs remained in the primed state if they remained in primed culture medium, and there was no significant difference in the expression of Fgf5, Pou5f1(Oct4), Nanog, Esrrb and Sox2 (Additional file 1: Fig. S2B, C). This suggests that Klf4 expression alone is insufficient to bring about the PNT. We then evaluated the PNT efficiency in three culture media: 15%FBS + 2i + LIF (serum + 2i + LIF medium, primed ESC culture medium), N2B27 + 2i + LIF (naïve mouse ESCs culture medium), and iCD1 (a high-efficiency somatic reprogramming medium) [30]. Comparatively, GFP + cells emerged earlier in iCD1 medium (1.8% at day 3), compared to 0.01/0.04% for the other media (Additional file 1: Fig. S2D, E). Consequently, we used iCD1 for the subsequent PNT experiments. iCD1 was also more efficient in activating the expression of pluripotent marker genes such as Sox2 and Esrrb which are known to remodel the chromatin structure during PNT [20, 30] (Additional file 1: Fig. S2F). PCA of the RNA-seq time course during PNT showed that the cells appeared to traverse an alternative path back to naïve ESCs, and did not retrace the NPT (Fig. 1B). Additionally, there was a spike in c-Jun expression in the PNT day 3 cells (Fig. 1D), which is likely due to cell heterogeneity as the PNT is an inefficient process. c-Jun expression is up-regulated at day 3 but is ultimately silenced in the resulting reverted iPSCs (Fig. 1D). These results suggest that the iCD1 medium is suitable for the conversion of primed EpiSCs to naïve ESCs.

To explore the impact of c-Jun on the PNT, we next performed PNT experiments on the c-JunKO EpiSC-like cells in iCD1 medium and measured the proportion of GFP positive cells at day 3 (D3) as a readout of PNT efficiency. Interestingly, we found that knock out of c-Jun significantly promoted the conversion of primed EpiSCs into naïve iPSCs, as measured by the number of GFP positive cells (2.7% versus 13.7% in the c-JunKO cells; Fig. 1G). Counting the resultant GFP + colonies indicated the conversion efficiency of c-JunKO EpiSC-like cells to ESCs was ~ 4 times higher than WT EpiSC-like cells on day 3 (Fig. 1G, H). Overall, these results suggest that c-Jun acts as a one-way valve to enable the PNT. In ESCs, c-Jun is not expressed and is not required for the NPT or EpiSCs, yet when the cells went through the PNT the loss of c-Jun drastically increased the efficiency of this conversion.

### c-Jun N-terminal kinase inhibitor promotes the primed to naïve transition

**Fig. 2:**
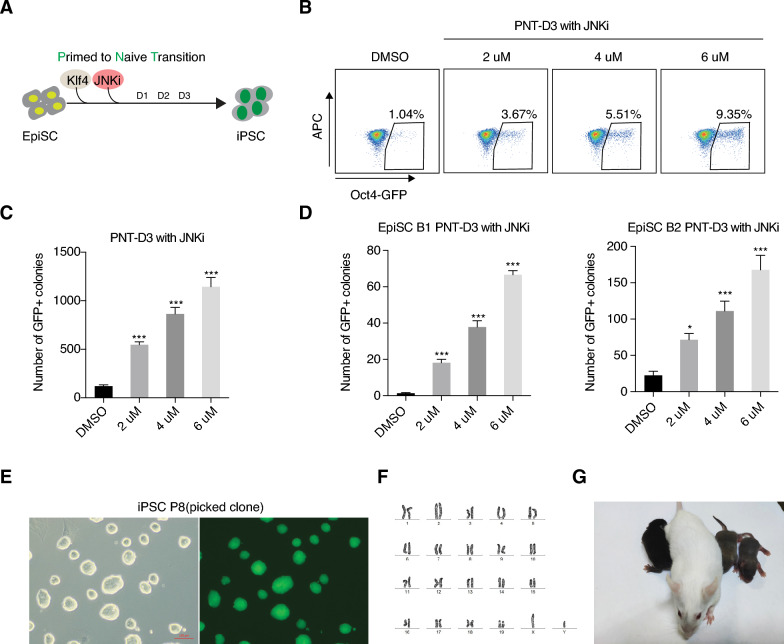
c-Jun N-terminal Kinase inhibitor facilitates primed to naïve Transition. **A**. Work model shows JNK inhibitor (SP600125) in primed to naive transition (PNT). **B**, Flow cytometry analysis represent reprogramming efficiency increased with a higher dose of JNKi. **C**, **D**. A dose-response graph for JNKi in promoting primed to naive transition. This experiment was performed with three different EpiSC cell lines called EpiSC B4 (this cell line was named EpiSC in the text, and used for this study), EpiSC B1, EpiSC B2. JNKi was used with 3 doses for 3 days during primed to naive transition (PNT) and scored for GFP positive colonies. Data are from 6 biological replicates in 2 independent experiments and shown as the mean ± SEM. *** indicates P value < 0.001, one-way ANOVA with Sidak correction between the DMSO and the dosing groups. **E**. Phase and GFP images of iPSC. Scale bar = 100 μm. **F**. Karyotypes of iPSC. **G**. Chimeric mice from iPSC.

Loss of c-Jun could dramatically improve the efficiency of the PNT. We next explored if small-molecule inhibition of upstream factors of c-Jun could also promote the PNT. We used a JNK inhibitor (JNKi, SP600125), which can indirectly inhibit c-Jun activity, to validate the negative function of c-Jun in PNT (Fig. 2A). Indeed, our results showed that the PNT was improved by JNKi in a dose-dependent manner, reaching almost 10% GFP + cells and ~ 1200 Oct4-GFP-positive colonies from 12000 starting cells on day 3 (D3) (Fig. 2B, C). A similar dose-dependent effect of JNKi could be seen when using two further independent lines of EpiSCs (B1 and B2) (Fig. 2D). The resulting iPSCs had normal naive morphology (Fig. 2E), karyotype (Fig. 2F), and were capable of generating chimeras with germline transmissions (Fig. 2G). These results suggest that inhibition of JNK phenocopies c-Jun KO in PNT.

Meanwhile, we found knock out other AP-1 family members such as Fosl2 also enhanced iPSCs formation (Additional file 1: Fig. S2G). On the contrary, PNT process was repressed when c-Jun was highly expressed in primed EpiSC (Additional file 1: Fig. S2H, I). Together, these results indicate that c-Jun is induced, but not needed for the naïve to primed transition or in EpiSCs. However, c-Jun functions as a barrier to the primed to naïve transition.

### c-Jun binds to closed naïve enhancers in EpiSCs

**Fig. 3:**
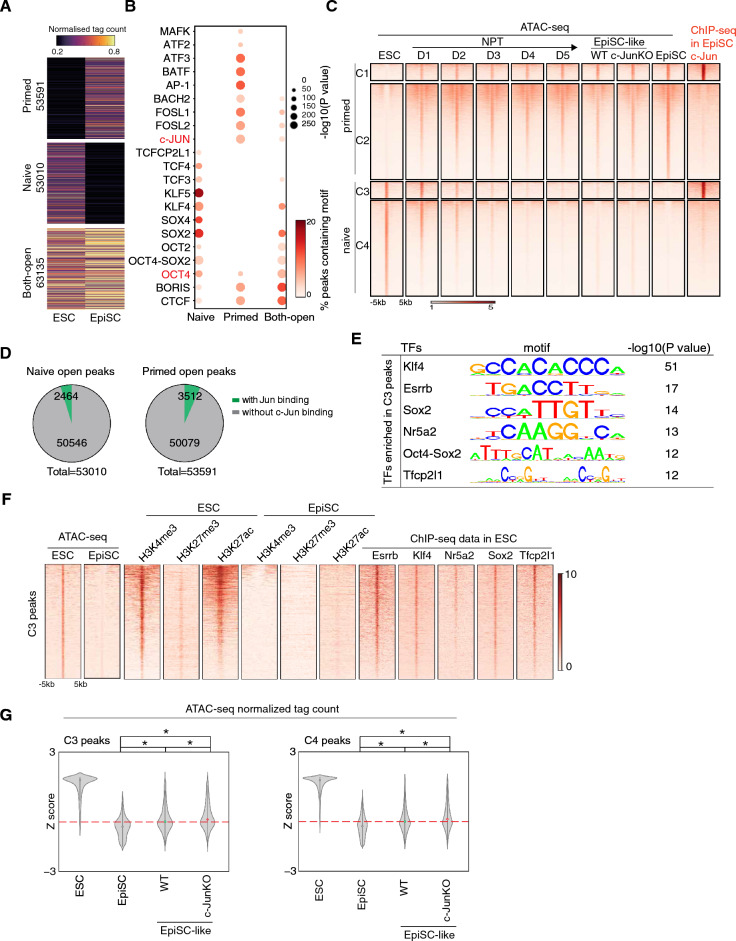
c-Jun bind to closed naïve enhancers in EpiSCs. **A**. ATAC-seq datasets from mouse EpiSC and ESC presented as open or closed state as described [9]. Top scale indicates normalized tag count in color shades. Note: primed for loci open in EpiSC; naive for loci open in ESC; Bothopen as open loci in both ESC and EpiSC. **B**. TF motifs enriched at least 1.5-fold higher than background and P value less than 0.01 for each category of ATAC-seq peaks defined in panel **A**. *P value < 10–20 from HOMER. **C**. Heatmap shows naive and primed loci closed and open during naive to prime transition in vitro at different time points. D indicates day of the differentiation process. Naive and primed ATAC-seq peaks defined in **A**. And c-Jun ChIP-seq data was performed in EpiSC, which divide naive and primed peaks into four groups. **D**. Pie chart presenting the number of peaks c-Jun occupied in primed and naive loci. **E**. Motif analysis showing pluripotent transcription factors that enriched in C3 peaks (peaks defined in **C**). **F**. Heatmap of sequence read density for ATAC-seq and H3K27ac, H3K27me3, H3K4me3 data in ESC and EpiSC signal on C3 peaks and pluripotency transcription factors Esrrb, Klf4, Nr5a2, Sox2 and Tfcp2l1 binding profile in C3 peaks. Sequenced reads ranked by mean signal strength. Windows are centered on the ATAC-seq pea summit. Each row of the heatmap is a genomic locus. The ChIP-seq data of H3K27me3 and H3K4me3 in ESCs were taken from GSE80280 [51], the ChIP-seq data of H3K27ac in ESCs was taken from GSE98404 [9] and the ChIP-seq data of H3K27me3, H3K27ac, H3K4me3 were taken from GSE57407 [52]. The ChIP-seq data of Sox2 and Klf4 were taken from GSE90893 [8], the ChIP-seq data of Esrrb and Tfcp2l1 were taken from GSE11431 [53] and the ChIP-seq data of Nr5a2 was taken from GSE1901 [54]. **G**. Violin plots for the normalized ATAC-seq tag density for all peaks in C3 and C4 groups. Data were converted to a Z score to the emphasis change. Data were converted to a Z score based on the row-wise SD for each peak. P value was calculated by the Mann-Whitney U test. * means P < 0.0005.

To explore the mechanism of how c-Jun impairs the PNT, we began by mapping the chromatin accessibility landscapes of both naïve ESCs and primed EpiSCs using ATAC-seq [31, 32]. 63135 peaks remained open in both cell types, but there was a surprisingly large number of ESC-specific (53010 peaks) or EpiSC-specific peaks (53591 peaks) (Fig. 3A). DNA motif enrichment analysis indicated that ESCs and EpiSCs had distinct transcription factor (TF) motifs associated with open chromatin in both states (Fig. 3B). As expected, OCT4 was common between the two cell types, including the compound OCT4-SOX2 motif that is instrumental in controlling the pluripotent state [33]. However, SOX, and especially KLF motifs, were specific to ESCs, whilst BACH, ATF and especially AP-1 family motifs were enriched in the EpiSCs (Fig. 3B). Indeed, the motif enrichment in naïve ESCs closely mirrored known regulatory programs, for example, TFCP2L1 and KLF motifs were specific to naïve, and when Tfcp2l1 and Klf5 are overexpressed they can drive EpiSCs to naïve ESCs in a PNT [34, 35]. Intriguingly, considering that c-Jun has no apparent function in EpiSCs, the AP-1 family of TF motifs (FOSL1, FOSL2, c-JUN) were enriched in primed-specific open chromatin (Fig. 3B). This enrichment of AP-1 family motifs led us to hypothesize that despite their apparent lack of function in EpiSCs, they may nonetheless be important in controlling the primed state.

To explore this, we performed ChIP-seq for c-Jun in EpiSCs (Additional file 1: Fig. S3A) and analyzed the binding sites with GREAT [36]. GREAT analysis reveals that c-Jun was bound to genes involved in normal cellular activity, such as apoptotic signaling, mitochondrial membrane organization, and adherens junction organization (Additional file 1: Fig. S3B). Unexpectedly, c-Jun was enriched at naïve-specific genes that are silenced in EpiSCs, for example, c-Jun was bound close to the genes, Dppa5a, Zfp42, and Esrrb (Additional file 1: Fig. S3C, D). Indeed, when we compared c-Jun binding sites to chromatin-accessible dynamics during NPT, we identified four groups: naïve closed but primed open with c-Jun bound (C1, 3521 peaks); naïve closed but primed open without c-Jun bound (C2, 50079 peaks); naïve open but primed closed and with c-Jun bound (C3, 2464 peaks); and naïve open but closed in primed, and without c-Jun (C4, 50546 peaks) (Fig. 3C, D). The most interesting group was C3, which had a distinctive epigenetic pattern: these loci were open in ESCs and were marked by enhancer-associated histone modifications H3K4me3, H3K27ac (Fig. 3F). However, in EpiSCs, these same loci are bound by c-Jun, their chromatin is closed and they have lost associated enhancer histone marks (Fig. 3C–F). This suggests those loci function as naïve-specific enhancers, that are silenced during NPT (Fig. 3C–F). Indeed, C3 loci are rapidly closed during the NPT as early as day 2 (Fig. 3C). Gene ontology analysis agreed with this designation and assigned the genes in C3 as related to transcription, cell proliferation and somatic stem cell population maintenance (Additional file 1: Fig. S3E). When we performed motif discovery on the C3 loci, motifs for many naïve-specific transcription factors (TFs): Klf4, Esrrb, Sox2, Nr5a2, and Tfcp2l1 were enriched (Fig. 3E). Analysis of ChIP-seq for these naïve-specific TFs in ESCs indicated that the C3 group loci are bound by all five of these TFs in ESCs (Fig. 3F). These results indicate that C3 loci are naive-specific enhancers near naïve-specific genes, that are bound by c-Jun in EpiSCs and are silenced.

We next looked if the loss of c-Jun altered chromatin at C3 in the c-JunKO EpiSC-like cells. Surprisingly, although consistent with the phenotype that c-Jun has minimal effects on EpiSCs, c-Jun deficiency had only a minimal impact on C3 loci closure during NPT (Fig. 1B). There was however a significant (if modest) increase in chromatin accessibility in the c-JunKO EpiSC-like cells (red dash line, Fig. 3G). This suggests that the C3 loci may be slightly more prone to opening, which may help explain why the loss of c-Jun promotes the PNT.

### c-Jun inhibits naïve loci opening during primed to naïve transition

**Fig. 4:**
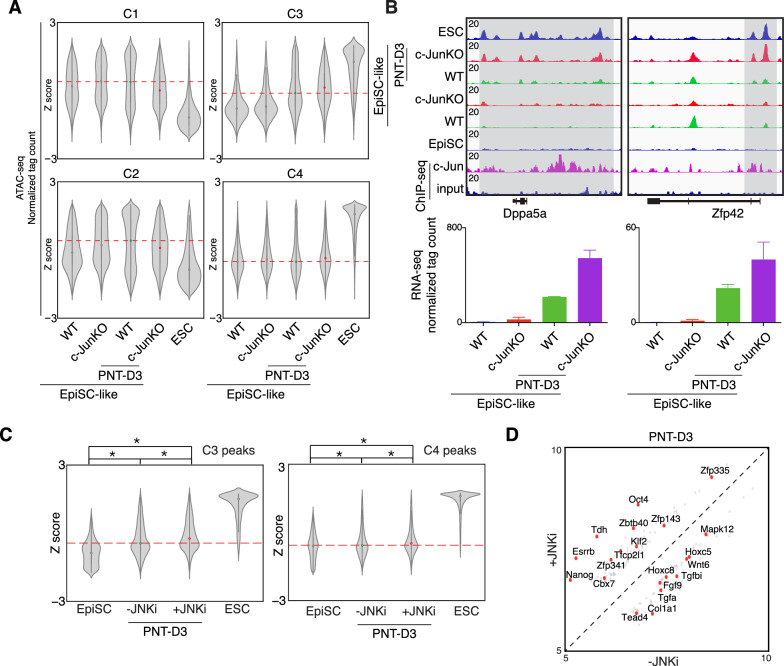
c-Jun represses PNT by maintain the closed naïve enhancers. **A**. Violin plots for the normalized ATAC-seq tag density for all peaks within the indicated primed (C1 and C2) and naive (C3 and C4) groups in WT EpiSC-like, c-Jun knock out EpiSC-like, PNT-D3 WT EpiSC-like, PNT-D3 c-Jun knock out EpiSC-like and ESC. D = day. Data were converted to a Z score based on the row-wise SD for each peak. **B**. Selected genome views for the ATAC-seq data and c-Jun ChIP-seq data in EpiSC, WT EpiSC-like, c-Jun knock out EpiSC-like, PNT-D3 WT EpiSC-like, PNT-D3 c-Jun knock out EpiSC-like and ESC. D = day. Genome views: Dppa5a (chr9:78,363,039–78,377,161), Zfp42 (chr8:43,293,267–43,309,209). The RNAseq expression values for the respective genes is shown in the bar plot below the genome view. RNA-seq data are from 2 biological replicates, expression units are in normalized tag counts. **C**. Violin plots for the normalized ATAC-seq tag count for the ATAC-seq data for C3 peaks, for EpiSC, PNT-JNKi-D3, PNT + JNKi-D3 and ESC. Data were converted to a Z score based on the row-wise SD for each peak. P value was from a Mann–Whitney U test. * means P < 0.0005. **D**. Scatterplot showing the difference between transcriptional profiles with or without JNKi at day 3 (D3) in PNT. JNKi promotes naive marker genes Nanog, Esrrb, Tfcp2l1 and Klf2 activation during PNT

To directly test the function of c-Jun in the primed to naïve transition (PNT), we analyzed the transcriptome and found that c-Jun deficiency promotes the up-regulation of 3746 genes during the PNT, amongst which 1688 genes are highly expressed in ESCs, and activated in c-Jun knock out cells at PNT day 3 (D3) (Additional file 1: Fig. S4A, B). Gene ontology analysis showed that those genes are involved in transcription, histone modification and stem cell population maintenance, and inner cell mass cell proliferation, and includes naïve-specific or embryonic-specific genes such as Dppa5a, Zfp42, Klf4, Gdf3, Kdm3a, Kdm5b, Sap30, Sall4 (Additional file 1: Fig. S4C, D). When we looked at chromatin accessibility dynamics, we found that c-Jun deficiency also promoted the opening of naïve-related loci, especially amongst the C3 group (Fig. 4A). For example, enhancers close to the Dppa5a and Zfp42 genes undergo considerably faster opening in the c-JunKO cells, compared to the wild type cells at day 3 (D3) (Fig. 4B). These changes in chromatin accessibility were accompanied by similarly rapid inductions of gene expression (Fig. 4B).

As we have shown that JNKi could promote PNT efficiency (Fig. 2), we also performed ATAC-seq in the JNKi-treated cells. The ATAC-seq data shown that JNKi also accelerate the opening of the C3 peaks (Fig. 4C). Consistently, JNKi activates the expression of Esrrb, Tfcp2l1, Klf2, Nanog in advance of the untreated PNT cells (Fig. 4D). These results indicate that loss of c-Jun or inhibition of JNK leads to a more rapid opening of chromatin at naïve-specific enhancers, and especially at the C3 group of genomic loci.

### Esrrb promotes PNT by opening c-Jun locked naïve loci

Our data suggest that c-Jun is required to suppress a class of naive-specific enhancers in EpiSCs, and the loss of c-Jun leads to priming of those loci and more rapid opening and gene expression in the PNT. To explore this further, we looked at the C3 loci, which were also enriched with the naïve-specific TFs Klf4, Esrrb, Nr5a2 and Tfcp2l1 (Fig. 3E, F). These four TFs have been implicated in earlier reports to mediate the PNT [20, 23, 24, 34]. Thus, we hypothesized that the loss of c-Jun primed the C3 group of loci and allowed easier access for these naïve-specific TFs. We co-transfected PiggyBac (integration-capable) plasmids bearing one of Esrrb, Klf4, Klf2, Nr5a1, Sox2 or Tfcp2l1 into EpiSCs, and then performed PNT. PNT was quantified by flow cytometry of the number of GFP + cells on day 3 (D3). Of the factors we transfected, Esrrb, Klf4, Klf2 and Nr5a1 could improve PNT, albeit to varying degrees of efficiency (Additional file 1: Fig. S5A, B). Interestingly, Esrrb could accelerate the PNT, and up to 15% of cells were Oct4-GFP-positive by day 3 (D3) (Additional file 1: Fig. S5B). Conversely, transfection of Sox2 and Tfcp2l1 could not improve the PNT (Additional file 1: Fig. S5B). To help explain the apparent differences in efficiency between the transgenes, we generated ATAC-seq data for all 6 factors during the PNT, and looked at the chromatin accessibility of C3 peaks at day 3 (PNT-D3). We found that, in agreement with the PNT efficiencies, Esrrb promoted the opening of C3 peaks more efficiently, while the others transgenes were less capable or were inefficient (Additional file 1: Fig. S5C). Overall, these results suggest that the loss of c-Jun primes naïve loci for opening, which leads to accelerated chromatin changes in combination with naïve-specific TFs (Additional file 1: Fig. S5D).

### c-Jun regulates chromatin accessibility in EpiSCs

**Fig. 5:**
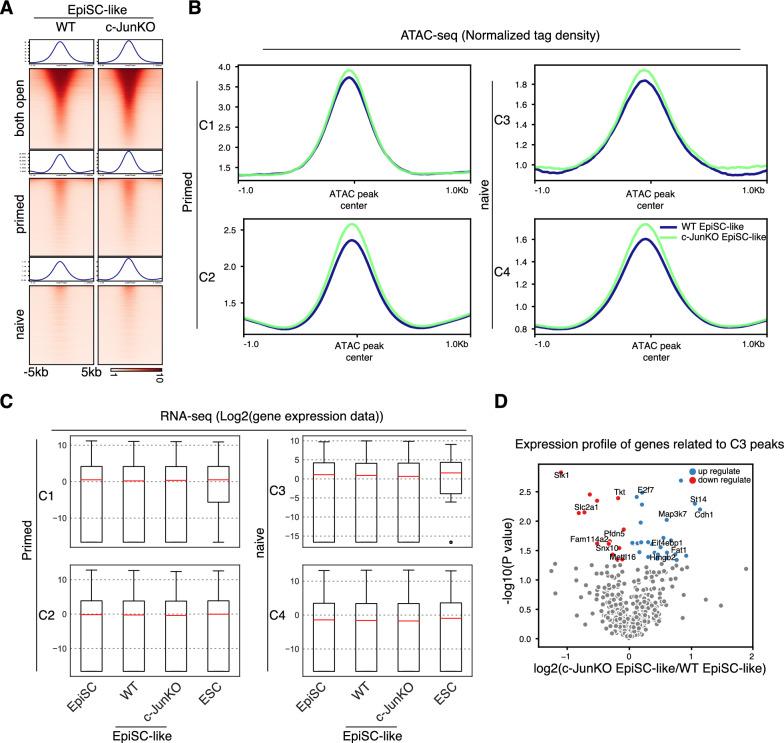
c-Jun regulates chromatin accessibility in EpiSCs. **A**. Heatmap of sequence read density for ATAC-seq data in EpiSC-like and c- Jun knock out EpiSC-like signal on both open, primed open and naive open category defined in Fig. 3A, ranked by mean signal strength. Windows are centered on the ATAC-seq peak summit. Each row of the heatmap is a genomic locus. **B**. Pileup of ATAC-seq read density at the naive and primed peaks in EpiSClike and c-Jun Knock out EpiSC-like. **C**. Boxplot of the expression level for all genes with a TSS within 10 kb of an ATAC-seq peak in C1 ~ C4 loci. Data were converted to a log2 value for each gene. **D**. Volcano plot show the expression of the genes related to C3 peaks, mapping the high expressed genes in EpiSC-like (left) and high expressed genes in c- JunKO EpiSC-like (right)

As we have shown c-Jun is not needed for the NPT, but it impedes the PNT and chromatin is more accessible in EpiSC-like cells when c-Jun was knocked out (Figs. 3G, 4A). This suggests that c-Jun might regulate EpiSC cell fate by locking naïve-specific enhancers. To study the mechanistic role of c-Jun in EpiSCs, we analyzed c-Jun ChIP-seq data in EpiSCs and found that the distribution of c-Jun appears to mainly localize at intragenic (39%) or intergenic (37%) regions at with only 16% at promoter regions. With a combined ~ 76% outside of coding regions [10], we hypothesized that c-Jun may participate in the regulation of chromatin dynamics in EpiSCs. However, the genome-wide pattern of chromatin accessibility was correlated between c-JunKO EpiSC-like cells, WT EpiSC-like cells and EpiSC (Additional file 1: Fig. S1D). When we looked at the accessibility of naïve and primed peaks in the c-JunKO EpiSC-like cells and WT EpiSC-like cells (peaks defined in Fig. 3A), we found the accessibility increased in both primed and naive peaks when c-Jun was knocked out (Fig. 5A, B). Among the C1 ~ C4 peaks, C1 and C3 were bound by c-Jun and the chromatin accessibility was elevated in the c-Jun knockout. This was especially evident in the C3 peaks which we previously identified as naïve-specific and bound by c-Jun (Figs. 3C, 5B, top-right). Meanwhile, C2 and C4 loci, which were not bound by c-Jun (Fig. 3C), also shown elevated levels of accessible chromatin in c-JunKO EpiSC-like cells (Fig. 5A, B). These data suggest that c-Jun is regulating chromatin at a specific subset of naïve-specific loci, and the loss of c-Jun leads to incomplete chromatin closure.

We next looked at the impact of the loss of c-Jun and changes in chromatin on gene expression. At the transcriptome level, we overlapped the genes annotated within 10 kb of a loci in C1 ~ C4 loci and shown similar expression pattern in EpiSC, EpiSC-like cells and c-JunKO EpiSC-like cells (Fig. 5C and Additional file 1: Fig. S1B). When detailed analysis the gene related to C3 locus, less genes were activated in c-Jun knockout EpiSC-like cells, such as Cdh1, E2f7 and Fat1 were up-regulated when c-Jun defect (Fig. 5D). These results indicate that c-Jun is required to maintain closed chromatin at a class of naïve-specific genes. Loss of c-Jun induces leaky expression of these genes which helps potentiate the EpiSCs for the PNT.

**Fig. 6:**
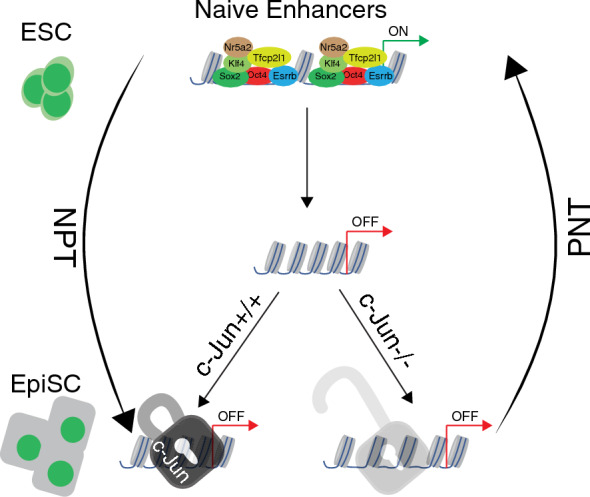
c-Jun functions as a lock to maintain closed naïve enhancers and represses the PNT. A model depicting the chromatin accessibility dynamics in naive to primed transition (NPT) and primed to naive transition (PNT). During NPT naïve enhancers were closed and bound by c-Jun. c-Jun functions as a lock to keep the naive enhancers in a closed state. However, if c-Jun is removed, the lock is loosened and the closed naive enhancers are primed and ready to recruit key naive-specific TFs to reactivate the naive gene regulatory network during the primed EpiSC to naive ESC transition

Collectively, our data indicate that c-Jun plays a stabilizing role in locking the silenced state of naïve loci in the primed state, and this helps block the primed to naïve transition and maintain EpiSCs cell identity (Fig. 6).

## Discussion

c-Jun is a key somatic factor that is expressed at low levels during early embryogenesis and is upregulated as cells commit to a somatic state. We have previously shown that c-Jun acts as a barrier to somatic cell reprogramming [4]. Here, we show that c-Jun can also block the primed to naïve transition (Fig. 6). Naïve cells roughly correspond to the early ICM, whilst primed cells correspond to the later epiblast, although the match is not perfect [16]. In mouse development, these two stages are only a few days apart but they are accompanied by significant changes in chromatin and transcriptome organization [37]. Many of these changes appear to be mimicked in the naïve ESCs and the primed EpiSCs [16, 38]. Crucially, whilst the NPT mimics a natural developmental pathway, the PNT is an artificial conversion with no in vivo developmental mimic. Here, we show that c-Jun erects an epigenetic barrier to the PNT, but is permissive to the NPT. Mechanistically, we show that c-Jun has no role in the NPT, but in the primed EpiSCs it is bound to naive-specific chromatin loci and acts to keep the chromatin closed at these loci (Figs. 3C, 5D). Loss of c-Jun through inhibition or knockout is not alone capable of initiating the PNT (Figs. 1H, 2B), but it does potentiate the cells for more efficient PNT as those naïve-specific loci now have more accessible chromatin, leaky RNA expression, and naïve-specific TFs can be more readily recruited to these loci to reactivate the naïve gene expression program. Interestingly, we also found that different EpiSC cell line showing different reprogramming efficiency, suggest a heterogeneity between EpiSC cell lines (Fig. 2C, D).

The finding that deletion of c-Jun activates silenced naïve enhancers and thus accelerates the primed to naïve transition suggests a direct repressive function of c-Jun (Fig. 4A). c-Jun has also been reported as a barrier to induce human stem cell differentiation into definitive endoderm (DE) and the repression of c-Jun with JNKi improves DE generation [5]. However, JNKi has also been reported to promote primed human ESCs reprogramming to naïve hESCs [39, 40], which is somewhat contradictory to the finding here, although this may reflect the confusing status of human naïve cells [17] or species-specific differences.

In chromatin dynamics, c-Jun has both activatory and inhibitory function that is context-dependent. As in the reprogramming of somatic cells into iPSCs, the c-Jun/AP-1 motif is enriched in active somatic enhancers [8, 9], and high expression of c-Jun can maintain the accessibility of somatic enhancers [9]. Ultimately, this suggests that c-Jun can have powerful activatory action on gene expression, however, there is also evidence for a repressive role for c-Jun. When c-Jun N-terminal phosphorylation is blocked by JNK inhibitors, c-Jun can recruit Mbd3/NuRD to AP-1 target genes and thus silence gene expression [12]. Overall, a detailed understanding of the activatory and repressive functions of c-Jun and how it modulates chromatin accessibility will shed light on why c-Jun is a crucial regulator of cell fate decisions and tumorigenesis [41–43].

Overall, our work shows how c-Jun can erect an epigenetic barrier that blocks the in vitro cell type conversion or primed EpiSCs to naïve ESCs. c-Jun helps maintain closed chromatin at naïve-specific genes, to impair the backward reversion of primed cells back to the naive state and is presumably one of several systems that overlap to block the PNT [20, 23, 24, 34]. Consequently, c-Jun helps construct a one-way developmental valve to control the direction of cellular development.

## Materials and methods

### Cell culture

HEK293T cells were cultured in high glucose DMEM supplemented with 10% fetal bovine serum (FBS, NATOCOR), GlutaMAX (GIBCO) and non-essential amino acids (NEAA, GIBCO), Mouse ESCs were cultured in 0.1% gelatin-coated plates with naïve N2B27 + 2iL medium (50% (v/v) DMEM (Hyclone), 50% (v/v) KnockOut DMEM (GIBCO), N2 (GIBCO), B27 (GIBCO), NEAA (GIBCO), GlutaMAX (GIBCO), PD0325901 (1 μM, In house-synthesized), CHIR99021 (3 μM, In house-synthesized), LIF (10 ng/ml, In house-synthesized)). HEK293T were purchased from ATCC (CRL-1126). mESCs were derived in-house. All of the cell lines have been confirmed as mycoplasma contamination-free with the MycoAlert Mycoplasma Detection Kit (Lonza, LT07-318).

### ESC derivation and culture

ESCs were derived from E3.5 mouse embryos from 129 female mice crossing male OG2 mice. Naïve ESCs were maintained in serum coated wells, and were removed and then washed the dish three times with DPBS before seeding naïve ESCs onto fresh wells. N2B27 + 2iL medium which comprised KnockOut DMEM (Gibco)/high glucose DMEM (Hyclone) (1:1) with N2 (200 × , Gibco), B27 (100 × , Gibco), NEAA (100 × , Gibco), GlutaMAX(100 × , Gibco), 0.1 mM 2-mercaptoethanol, 1000 U/ml LIF, 3 uM Chir99021 and 1 uM PD0325901 was used for naïve ESCs culture. ESCs were passaged every two to three days as single cells with Trypsin–EDTA (0.25%).

### EpiSCs derivation and culture

EpiSCs were derived from ~ E5.5 mouse embryos from 129 female mice crossed with male OG2 mice. EpiSCs were maintained on feeders in an optimized KFN medium which comprised KnockOut DMEM-F12 (Gibco)/Neurobasal (Gibco) (1:1) with N2 supplement (200 × , Gibco), B27 supplement (100 × , Gibco), NEAA (100 × , Gibco), GlutaMAX (100 × , Gibco), Lactalbumin hydrolysate (1 mg/ml, Gibco), 100 mM 2-mercaptoethanol, 20 ng/ml Activin A and 20 ng/ml bFGF. For feeder-free culture, EpiSCs were maintained in serum-coated wells, and serum was removed and then the well was washed three times with DPBS before replating the EpiSCs. EpiSCs were also grown feeder-free in FN medium, comprised of F12 (Ham’s F12, Gibco)/Neurobasal (Gibco) (1:1) with N2 (200 × , Gibco), B27 (100 × , Gibco), NEAA (100 × , Gibco), GlutaMAX (100 × , Gibco), BSA (1 mg/ml, Gibco), 100 mM 2-mercaptoethanol, 20 ng/ml Activin A and 20 ng/ml bFGF was used for EpiSCs culture. EpiSCs were passaged every 2 to 3 days as small clumps with 1.5 mg/ml Collagenase IV (Invitrogen 17104–019) or as single cells with Accutase (Sigma-A6964). For single-cell passaging, 10 µM Y-27632 was added in the medium on the first day to maintain high levels of EpiSCs survival.

### Induced ESCs into EpiSC-like cells (NPT)

To differentiate ESCs into EpiSC-like cells [27, 28], ESCs were digested to single cells and replanted in serum serum-coated 6-well plate at a density of 1 × 10^4^ per well. After 8 h, the ESCs culture medium was removed, and cells were washed with DPBS twice carefully. FN medium-plus XAV939 (2 µM) and IWR-1 (2.5 µM) was used to convert ESCs to EpiSC-like cells. FN + X/I medium was changed every day, and EpiSC-like colonies emerged at days 4–5. EpiSC-like cells were cultured feeder-free in EpiSC medium (FN medium) and passaged every 2 to 3 days in the same way that EpiSCs were passaged.

### Generation of Klf4-transgenic EpiSCs (KEpiSCs) lines

To establish Klf4-transgenic EpiSCs lines, lentiviruses were prepared by transfecting pRlenti-Klf4-IRES-neo vector into HEK293T cell line using calcium phosphate method. EpiSCs were digested with Accutase and replanted in serum coated 12-well plate at a density of 3 × 10^4^ per well 24 h before infection. Lentiviruses supernatants supplemented with 4 μg/ml polybrene were used to infect EpiSCs. Fresh EpiSC culture medium was changed after 12 h’ infection. To select for stable Klf4-expression cell lines, 400 μg/ml G418 was applied for at least 2 weeks.

### Resetting EpiSCs into a naïve state (PNT)

To reset EpiSCs (including EpiSC-like) into a naïve ES-like cells, the EpiSCs were digested with Accutase and replanted in serum coated 24-well plate at a density of 1.2 × 10^4^ per well, and maintained in FN medium plus 10 µM Y-27632. After 24 h, the medium was replaced with iCD1 [30] medium containing JNK inhibitor SP600125 at the final concentrations of 2 μM, 4 μM and 6 μM. The medium was changed every day, and GFP positive colonies were analyzed under the microscopy at resetting day3.

### Generation of knock out ESCs

c-Jun-/- ESCs and EpiSCs were generated by CRISPR/Cas9, and pairs of sgRNA were used to deletion the target exons. sgRNA1: ggctgtgcgcagaagtttcg; sgRNA2: gacaaacttgagaacttgac.

### Generation of chimera mice

For the generation of chimaeras, reset iPSCs lines were injected into CD-1 blastocysts and followed by implantation into pseudopregnant CD-1 female mice. Chimeric mice could be identified by coat color. Germline transmission of the chimera mice was determined by breeding F2 mice to CD-1 mice. All of the animal experiments were performed with the approval and according to the guidelines of the Animal Care and Use Committee of the Guangzhou Institutes of Biomedicine and Health.

### Karyotype analysis

Cell cultures were prepared to give a 70 ± 80% confluence on the day of sampling. After 1–2 h incubation with fresh medium which was added a Colcemid solution at a final concentration of 0.02 mg/ml. Then the cells were washed in PBS, trypsinized and spun down. To obtain a single-cell suspension, the pellet was re-suspended in hypotonic solution (0.075 mol/L KCl) and left at 37 ℃ temperature for 18–20 min. After spinning and removing the hypotonic solution, 1 mL of ice-cold fixative (3:1 methanol: acetic acid) was added dropwise to the suspension, left at 37 ℃ temperature for 40 min and then spun down. Finally, the pellet was re-suspended in a final volume of 1 mL fixative. The cells were then dropped onto trypsin Solution (per 55 ml saline solution with 0.03 g Pancreatin) washed slides and stained with Giemsa. The number of chromosomes, as well as the presence of structural chromosomal abnormalities, was examined.

### Flow cytometry

Intermediate cells undergoing the PNT at day 3 were digested by 0.25% trypsin, and washed with PBS. The cells were resuspended in flow cytometry buffer (PBS with 2% FBS) and analyzed with Accuri™ C6 Plus (BD Biosciences). The FACS data was analyzed with FlowJo software.

### Immunobloting

Cells were collected and lysed in lysis buffer supplemented with protease inhibitor cocktail on ice for 10 min, then boiling the cells in 100 ℃ for 10 min. After centrifugation, the cell supernatants were subjected to SDS-PAGE and incubated with c-Jun and GAPDH antibodies. The following antibodies were used in the project: anti-c-Jun (CST no.9165, 1:3000), and anti-GAPDH (Bioworld, AP2063, 1:3000).

### Immunofluorescence

Cells growing on coverslips were washed 3 times with PBS, then fixed with 4% PFA for 30 min, and subsequently cell membranes were penetrated with 0.1% Triton X-100 and blocked with 3% BSA for 30 min at room temperature. Cells were then incubated with c-Jun antibody for 1 h. After 3 washes in PBS, followed by 1 h of incubation in secondary antibodies, cells were incubated in DAPI for 1 min. Then the coverslips were mounted on the slides for observation on a confocal microscope (Leica). The following antibody was used in this project: anti-c-Jun (CST no.9165, 1:100).

### Quantitative PCR and RNA-seq

Total RNAs were extracted by a chloroform-isopropanol method. For quantitative PCR, the first-strand cDNAs were synthesized with ReverTra Ace (Toyobo) and oligo-dT (Takara) and then performed on a CFX96 real-time system (Bio-Rad) with SsoAdvanced™ Universal SYBR^®^ Green Supermix (Bio-Rad). For RNA-seq, RNA-seq libraries were prepared following the instructions of VAHTS mRNA-seq V2 Library Prep Kit for Illumina (NR601, Vazyme). The libraries were quantified with Qubit and sequenced on a NextSeq 500.

### ATAC-seq

ATAC-seq was performed as previously described [31, 32]. Briefly, 50,000 cells were washed with 50 μl cold PBS and resuspended in 50 μl lysis buffer (10 mM Tris–HCl pH 7.4, 10 mM NaCl, 3 mM MgCl2, 0.2% (v/v) IGEPAL CA-630). The suspension of nuclei was then centrifuged for 10 min at 500 g at 4 °C, followed by the addition of 50 μl transposition reaction mix (25 μl TD buffer, 2.5 μl Tn5 transposase and 22.5 μl nuclease-free H_2_O) of Nextera DNA library Preparation Kit (96 samples) (FC-121-1031, Illumina). Samples were then PCR amplified and incubated at 37 °C for 30 min. DNA was isolated using a MinElute Kit (Qiagen). ATAC-seq libraries were first subjected to 5 cycles of pre-amplification. To determine the suitable number of cycles required for the next round of PCR, the libraries were assessed by quantitative PCR as described [32], and the libraries were then PCR amplified for the appropriate number of cycles according to the qPCR results. Libraries were purified with a Qiaquick PCR (Qiagen) column, and the libraries concentration were measured using a KAPA Library Quantification kit (KK4824) according to the manufacturer’s instructions. Finally, the ATAC libraries were sequenced on NextSeq 500 sequencing platform using a NextSeq 500 High Output Kit v2 (150 cycles) (FC-404-2002, Illumina) according to the manufacturer’s instructions.

### ChIP-seq

c-Jun ChIP was performed as described previously [4]. Every 1 × 10^9^ EpiSCs were fixed in 8.75 ml DMEM/F12 with 1% formaldehyde for 15 min at room temperature with rotation and then followed by the reaction with 0.125 M glycine. Cells were then lysed in ChIP-buffer A (50 mM HEPES–KOH, 140 mM NaCl, 1 mM EDTA (pH 8.0), 10% glycerol, 0.5% NP-40, 0.25% Triton X-100, 50 mM Tris–HCl (pH 8.0), and protease inhibitor cocktail) for 10 min at 4 °C. Samples were centrifuged at 1,400 g for 5 min at 4 °C. Pellets were lysed in ChIP-buffer B (1% SDS, 50 mM Tris–HCl (pH 8.0), 10 mM EDTA and protease inhibitor cocktail) for 5 min at 4 °C. The DNA was fragmented to 100–500 bp by sonication and then centrifuged at 12,000 g for 2 min. The supernatant was diluted with ChIP IP buffer (0.01% SDS, 1% Triton X-100, 2 mM EDTA, 50 mM Tris–HCl (pH 8.0), 150 mM NaCl and protease inhibitor cocktail). Immunoprecipitation was performed with 2 µg rabbit anti-c-Jun antibody (Abcam, ab31419) coupled to Dynabeads with proteinA/G overnight at 4 °C. Beads were washed, eluted and reverse cross-linked. DNA was extracted by phenol/chloroform for sequencing. The ChIP DNA library was constructed with VAHTS Universal DNA Library Prep Kit for Illumina (Vazyme Biotech, Nd604) according to the manufacturer’s instructions. The DNA libraries were quantified and tested by qPCR with positive primers to assess the quality of the library. Then, libraries were sequenced on an Illumina NextSeq 500 instrument using 75 bp paired-end reads.

### RNA-seq analysis

RNA-seq was processed as described in [9]. All sequencing data were mapped to the mm10 mouse genome assembly using bowtie2 with the options –very-sensitive. samtools was used remove low quality mapped reads with options view –q 35 and only unique reads were kept. We removed mitochondrial sequences using ‘grep –v ‘chrM’’. Biological replicates were merged, and peaks were called using dfilter [44] (with the settings: -bs = 100 –ks = 60 –refine –std = 5). BigWig files were produced using genomeCoverageBed from bedtools (scale = 10^7^/ < one sample’s total unique reads >) and then bedGraphToBigWig. Gene ontology and gene expression measures were called by first collecting all transcription start sites within 10 kb of an ATAC-seq peak, and then performing GO analysis with goseq [45], or measuring gene expression. Other analysis was performed using glbase [46]. Gene ontology analysis was performed using DAVID [47, 48] with default settings.

### Re-calling weak peaks from the ATAC-seq data

Peaks recalling is based on the method we previously described [9]. Briefly, when dfilter is used to discover peaks, as described above, it is generally conservative, and will not call a weak peak. Hence, we ‘re-call’ all peaks by measuring the sequence tag density in all ATAC-seq libraries, for all possible peaks in any other ATAC-seq library, irrespective of which library the peak was called in by dfilter [46]. Then we get a superlist of all possible peaks in any library, and based on our previous analysis we used an arbitrary minimum threshold of 0.2734 to filter out false peaks, if the ATAC-seq is below this value it is annotated as ‘closed’ and above ‘open’. This led to more accurate peak calling, as weak peaks rejected by dfilter could be recovered as a real peak. All downstream analysis is based on this new peak list.

### ChIP-seq data analysis

Reads from ChIP-seq experiments were mapped to the mouse genome (mm10) using Bowtie2 (-very-sensitive), as described for ATAC-seq data, and only the uniquely mapped reads were kept for further analysis. Peaks were called using MACS2 [49] software with the default parameters.

### Computational analysis of TF binding sites and open chromatin regions

Motif analysis was performed by HOMER [50] with default settings. Motifs were only kept if the P value was < 0.01 and (< percent of target > / < percent of background >) was > 1.5. Annotation of the ChIP-seq/ATAC-seq peaks/open chromatin to genes was performed by HOMER [50] using default settings. Gene ontology analysis of ChIP-seq binding or open chromatin was performed using GREAT [36] with default settings.

### Statistical analysis

For ATAC-seq data analysis, comparisons between two groups were made using Mann–Whitney U test. For the comparisons of reprogramming efficiency between two samples were made using a t-test.

### Supplementary Information

**Additional file 1: Figure S1.** The binding profile of c-Jun in EpiSC. A. Schematic shows the knockout targeting strategy for c-Jun. The CRISPR/Cas9 was applied to produce specific double-strand breaks and deletion c-Jun protein-coding sequence. B. Scatter plot showing the values of log2(FPKM) for each gene in EpiSC (Xaxis) versus the EpiSC-like cells (Y-axis) and c-JunKO EpiSC-like cells (Y-axis). C,D. Hierarchical clustering of the pairwise (Pearson) correlation between RNA-seq data and ATAC-seq data in ESC, naïve to primed transition time course samples (NPT-D1~NPTD5), EpiSC-like cells and EpiSC. Data were clustered based on a Euclidean distance matrix and complete linkage. **Figure S2.** Set up primed to naïve transition (PNT) system. A. Schematic diagram depicts the reprogramming process from primed to naive transition (PNT). B. Representative fields show EpiSC and kEpiSC (EpiSC transfected with Klf4). Klf4 is only expressed in kEpiSC shows below. C. qRT-PCR analysis for the expression of Klf4, Fgf5, Oct4, Nanog, Esrrb, Sox2 with two replicates in EpiSC, kEpiSC and ESC cell lines. D. Phase and GFP images of representative %15FBS+2i+Lif, N2B27+2i+Lif and iCD1 induced PNT at D3. D=day. Scale bar=100 μm. E. Flow cytometry analysis Oct4-GFP reporter of three reprogramming mediums. F. qRT-PCR analysis the pluripotent gene expression value under indicated culture medium. Data are from two biological replicates and shown as the mean±SEM. G, GFP positive colonies of WT kiEpiSCs and c-Jun-/- kiEpiSCs, Atf3-/- kiEpiSCs, Fosl2-/- kiEpiSCs induced with or without JNKi for 3 days. Data were from 4 biological replicates in 2 independent experiments and shown as the mean±SEM. ** indicates P value < 0.005, t test analysis between the knock out cell lines with WT kiEpiSCs. H, c-JUN was transfected into kEpiSC and then performed PNT experiment under iCD1 plus JNKi medium. D=day, Scale bar=200μm. I, Bar chart showing the percentage of cells GFP+ were significantly decreased during PNT when c-JUN was over-expressed. Data were from 12 biological replicates in 3 independent experiments and shown as the mean±SEM. *** indicates P value < 0.001, t test analysis between c-JUN OE and control groups. **Figure S3.** c-Jun ChIPseq in primed EpiSC. A. Reads of c-Jun ChIP-seq data mapped to two strands are treated separately to build two coverage density profiles. B. A gene ontology (GO) term enrichment analysis on c-Jun binding genes with GREAT (35). C. In primed EpiSC, c-Jun binding is correlated with pluripotency genes that are highly expressed in naïve ESC but downregulated in primed EpiSC. The density of c-Jun binding was then averaged using a moving average window to visualize the binding density of c-Jun across the gene changes. Fold Change > 1 was used to screen high expression genes between two samples. D. Selected genomic views of the ATAC-seq data and ChIP-seq data, are shown for the indicated naïve peaks: Dppa5a (chr9:78,366,097-78,376,829), Zfp42 (chr8:43,291,353-43,309,179), Esrrb (chr12:86,460,691-86,522,913). E. Gene ontolEogy analysis of genes related to C3 peaks. **Figure S4.** Knock out c-Jun activates pluripotent genes during primed to naïve transition. A. Venn diagrams showing the overlap genes between high expression genes in ESC and up-regulated genes in c-Jun knock out primed to naïve transition (PNT) day 3 cells. Fold Change > 1 was used to screen high expression genes between two samples. B. Violin plots of the expression level for overlap genes (1688) in panel A. Data were converted to a Z score based on the row-wise SD for each gene. C. Gene ontology analysis of the overlap genes (1688) in panel A. Analysis was performed using DAVID. D. Heatmap showing the expression of 16 selected pluripotency genes from the overlap genes (1688) in panel A. RNA-seq data is shown as log2(Fold Change) relative to expression in EpiSC. The value of Log2(Fold Change) was shown in each block. **Figure S5.** Esrrb promotes PNT by opening c-Jun locked naïve loci A. Esrrb, Klf4, Klf2, Nr5a1, Sox2 and Tfcp2l1 were cloned into PiggyBac plasmid and each of the six PiggyBac plasmids was transfected into EpiSC and then perform PNT experiment. The number of iPSC colonies were count at PNTD3. D=day. Scale bar=100 μm. B. Bar chart showing the percentage of cells GFP+ (GFP expressed from the Oct4-GFP, OG2 reporter) during primed to naïve transition (PNT) by the defined TFs. Data are from 4 biological replicates in 2 independent experiments and are shown as the means ± SEM. C. Average ATAC-seq read density at C3 peaks in ESC, EpiSC and cells of primed to naïve transition (PNT) induced by each of the six TFs at day 3 (PNTD3). D. Working model of the naive TFs in reprogramming primed to naïve transition (PNT). Naïve TFs were binding to the closed naïve enhancers which bind by cJun and opening the silenced naïve enhancers to activate the pluripotent program.

## Data Availability

The RNA-seq data, ChIP-seq data and ATAC-seq data used in this study have been deposited in the Gene Expression Omnibus (GEO) under accession number GSE127925, GSE127921 and GSE127920. All other relevant data are available from the corresponding author on request.
