# Supplementary material for: c-Jun as a one-way valve at the naive to primed interface
Source: Cell Biosci. 2023 Oct 14;13:191. doi: 10.1186/s13578-023-01141-0 (PMC10576270; doi:10.1186/s13578-023-01141-0)
Supplement: Supplementary file 1 — Additional file 1: Figure S1. The binding profile of c-Jun in EpiSC. A. Schematic shows the knockout targeting strategy for c-Jun. The CRISPR/Cas9 was applied to produce specific double-strand breaks and deletion c-Jun protein-coding sequence. B. Scatter plot showing the values of log2(FPKM) for each gene in EpiSC (Xaxis) versus the EpiSC-like cells (Y-axis) and c-JunKO EpiSC-like cells (Y-axis). C,D. Hierarchical clustering of the pairwise (Pearson) correlation between RNA-seq data and ATAC-seq data in ESC, naïve to primed transition time course samples (NPT-D1~NPTD5), EpiSC-like cells and EpiSC. Data were clustered based on a Euclidean distance matrix and complete linkage. Figure S2. Set up primed to naïve transition (PNT) system. A. Schematic diagram depicts the reprogramming process from primed to naive transition (PNT). B. Representative fields show EpiSC and kEpiSC (EpiSC transfected with Klf4). Klf4 is only expressed in kEpiSC shows below. C. qRT-PCR analysis for the expression of Klf4, Fgf5, Oct4, Nanog, Esrrb, Sox2 with two replicates in EpiSC, kEpiSC and ESC cell lines. D. Phase and GFP images of representative %15FBS+2i+Lif, N2B27+2i+Lif and iCD1 induced PNT at D3. D=day. Scale bar=100 μm. E. Flow cytometry analysis Oct4-GFP reporter of three reprogramming mediums. F. qRT-PCR analysis the pluripotent gene expression value under indicated culture medium. Data are from two biological replicates and shown as the mean±SEM. G, GFP positive colonies of WT kiEpiSCs and c-Jun-/- kiEpiSCs, Atf3-/- kiEpiSCs, Fosl2-/- kiEpiSCs induced with or without JNKi for 3 days. Data were from 4 biological replicates in 2 independent experiments and shown as the mean±SEM. ** indicates P value < 0.005, t test analysis between the knock out cell lines with WT kiEpiSCs. H, c-JUN was transfected into kEpiSC and then performed PNT experiment under iCD1 plus JNKi medium. D=day, Scale bar=200μm. I, Bar chart showing the percentage of cells GFP+ were significantly decreased during PNT [file 13578_2023_1141_MOESM1_ESM.docx]

Figure. S1 (Related to Figure 1)

**A B**


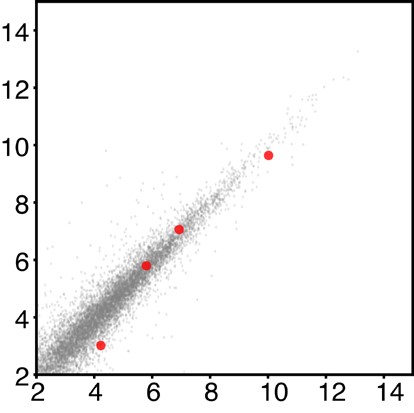


c-JunKO EpiSC-like

R=0.947

c-Jun

Fgf5

Nanog

Oct4


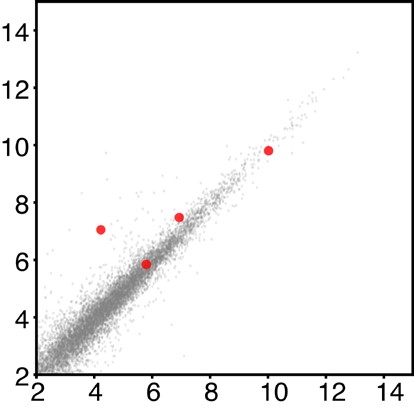


EpiSC-like

R=0.959

c-Jun

Fgf5

Nanog

Oct4

sgRNA1 sgRNA2

Exon

EpiSC-like

c-Jun

EpiSC EpiSC

**C D**

RNA-seq ATAC-seq

Correlation(pearson) Correlation(pearson)


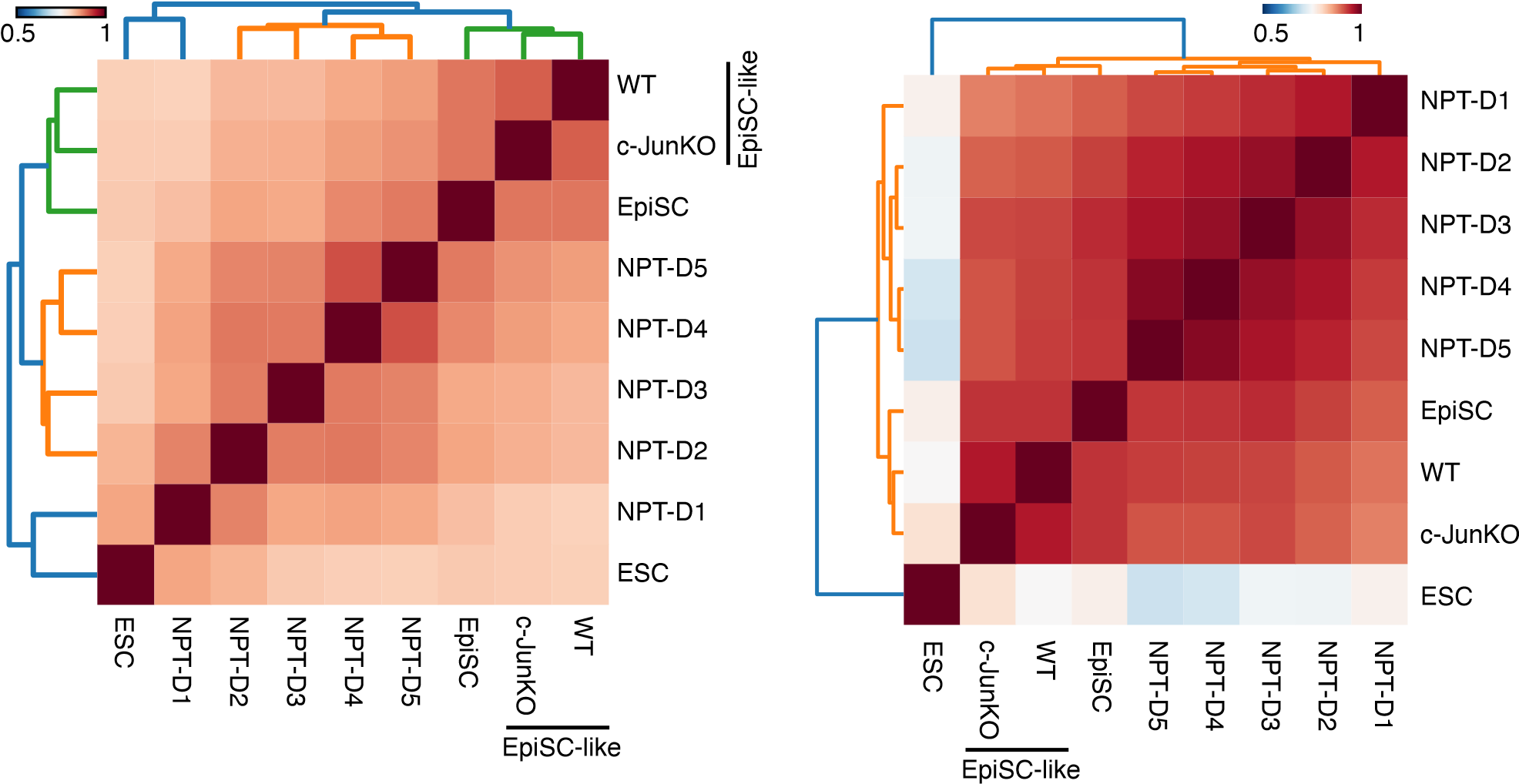


# S1. The binding profile of c-Jun in EpiSC

Schematic shows the knockout targeting strategy for c-Jun. The CRISPR/Cas9 was applied to produce specific double-strand breaks and deletion c-Jun protein-coding sequence.

**B.** Scatter plot showing the values of log2(FPKM) for each gene in EpiSC (Xaxis) versus the EpiSC-like cells (Y-axis) and c-JunKO EpiSC-like cells (Y-axis). **C,D.** Hierarchical clustering of the pairwise (Pearson) correlation between RNA-seq data and ATAC-seq data in ESC, naïve to primed transition time course samples (NPT-D1~NPTD5), EpiSC-like cells and EpiSC. Data were clustered based on a Euclidean distance matrix and complete linkage.

Figure S2 (Related to Figure 1)


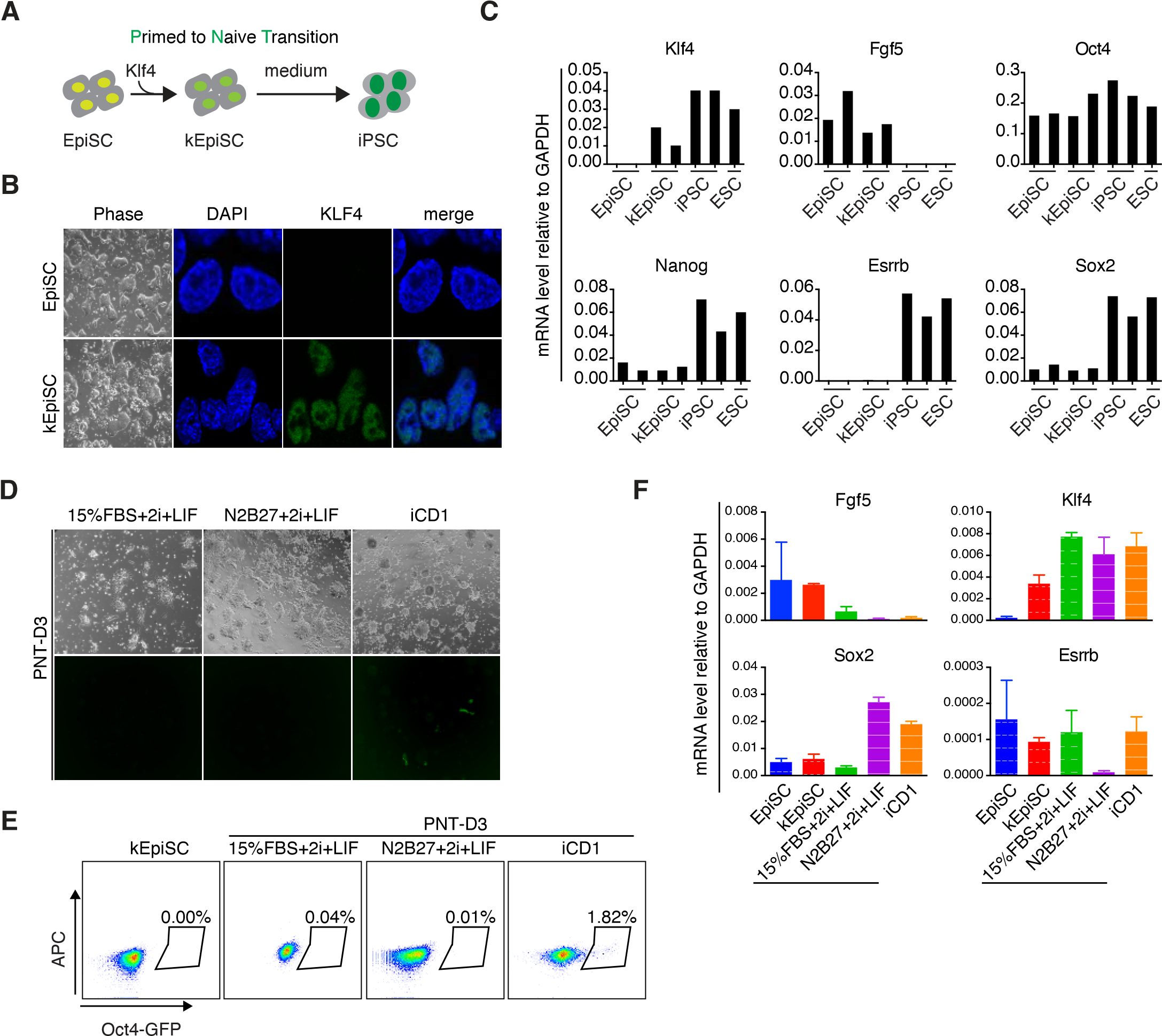
PNT-D3 PNT-D3

# G H I

iCD1

i

CD1

+

JNK

i

WT


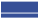


c-J

un

-

/

-


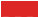


A

tf

3

-

/

-


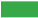


Fo

s

l2

-

/

-


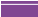


N

u

m

b

e

r

o

f

GF

P

+

c

oloni

e

s

0

10

0

20

0

30

0

40

0

**

**

**

**

kEpiSC-like D3

Control

OE c-Jun

0

5

10

15

Percent of cells GFP+

***

kEpiSC D3

iCD1+JNKi


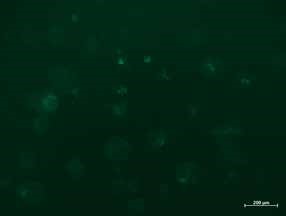

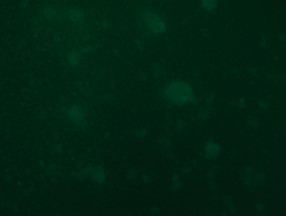


Control

OE c-Jun

iCD1+JNKi-D3


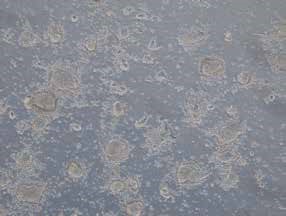

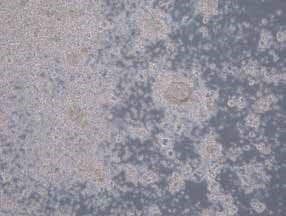


## S2. Set up primed to naïve transition (PNT) system

Schematic diagram depicts the reprogramming process from primed to naive transition (PNT).

1. Representative fields show EpiSC and kEpiSC (EpiSC transfected with Klf4).

Klf4 is only expressed in kEpiSC shows below.

1. qRT-PCR analysis for the expression of Klf4, Fgf5, Oct4, Nanog, Esrrb, Sox2 with two replicates in EpiSC, kEpiSC and ESC cell lines.
2. Phase and GFP images of representative %15FBS+2i+Lif, N2B27+2i+Lif and iCD1 induced PNT at D3. D=day. Scale bar=100 μm.
3. Flow cytometry analysis Oct4-GFP reporter of three reprogramming mediums.
4. qRT-PCR analysis the pluripotent gene expression value under indicated culture medium. Data are from two biological replicates and shown as the mean±SEM.

**G**, GFP positive colonies of WT kiEpiSCs and c-Jun-/- kiEpiSCs, Atf3-/- kiEpiSCs, Fosl2-/- kiEpiSCs induced with or without JNKi for 3 days. Data were from 4 biological replicates in 2 independent experiments and shown as the mean±SEM. ** indicates P value < 0.005, t test analysis between the knock out cell lines with WT kiEpiSCs.

**H**, c-JUN was transfected into kEpiSC and then performed PNT experiment under iCD1 plus JNKi medium. D=day, Scale bar=200μm.

**I**, Bar chart showing the percentage of cells GFP+ were significantly decreased during PNT when c-JUN was over-expressed. Data were from 12 biological replicates in 3 independent experiments and shown as the mean±SEM. *** indicates P value < 0.001, t test analysis between c-JUN OE and control groups.

Figure S3 (Related to Figure 3)

**A B**

Gene ontology analysis of c-Jun associated genes

c-Jun ChIP-seq in EpiSC regulation of intrinsic apoptotic signaling pathway

**0**

**2**

**0**

**4**

**0**

**6**

**0**

**8**

**0**

0.00

0.30

P

ercentage

f

or

w

ard tags

r

e

v

erse tags

Peak Model

mitochondrial membrane organization protein folding

regulation of mitochondrial membrane permeability regulation of membrane permeability adherens junction organization

negative regulation of intrinsic apoptotic signaling pathway organelle transport along microtubule

−600 600 cellular response to topologically incorrect protein

Distance to the middle

intrinsic apoptotic signaling pathway in response to DNA damage

-log10(binom P value)

**C D**


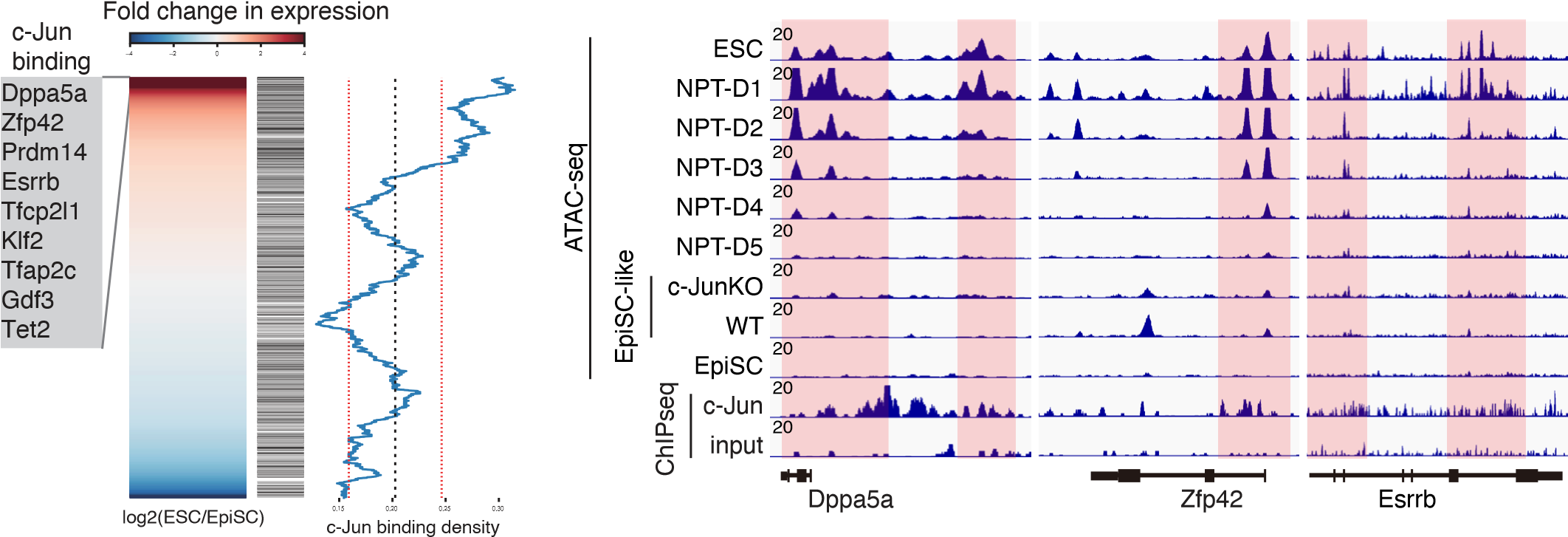


**E**

Gene ontology analysis of genes related to C3 peaks Transcription, DNA-templated

0

2

4

6

8

-

log10(P value

)

Actin cytoskeleton organization

Cell migration

Cell proliferation

Somatic stem cell population maintenance

**S3. c-Jun ChIPseq in primed EpiSC**

Reads of c-Jun ChIP-seq data mapped to two strands are treated separately to build two coverage density profiles.

1. A gene ontology (GO) term enrichment analysis on c-Jun binding genes with GREAT (35).
2. In primed EpiSC, c-Jun binding is correlated with pluripotency genes that are highly expressed in naïve ESC but downregulated in primed EpiSC. The density of c-Jun binding was then averaged using a moving average window to visualize the binding density of c-Jun across the gene changes. Fold Change > 1 was used to screen high expression genes between two samples.
3. Selected genomic views of the ATAC-seq data and ChIP-seq data, are shown for the indicated naïve peaks: Dppa5a (chr9:78,366,097-78,376,829), Zfp42 (chr8:43,291,353-43,309,179), Esrrb (chr12:86,460,691-86,522,913). **E.** Gene ontolEogy analysis of genes related to C3 peaks.

Figure S4 (Related to Figure 4)

**A B**

RNA-seq

0

Z score

1688

genes activated during PNT

3

-3

WT-rp1

WT-rp2

c-JunKO-rp1

c-Jun KO-rp2

PNT-D3

ESC-rp1

ESC-rp2

WT-rp1

WT-rp2

c-JunKO-rp1

c-JunKO-rp2

EpiSC-rp1

EpiSC-rp2

2195

2058

1688

EpiSC-like PNT-D3

log2(ESC/EpiSC) > 0 log2(c-JunKO/WT) > 0

EpiSC-like

**C D**

protein transport

cell cycle**_0.5_**


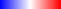


Log2(Fold Change)

-10

10


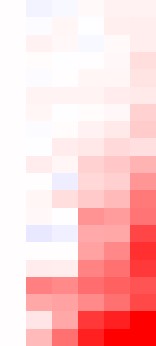


Dppa5a

Zfp42

Klf2

Klf4

Esrrb

Prdm14

Tfcp2l1

Tfap2c

Gdf3

Tet2

Sox2

Sall1

Gli2

Kdm3a

Ube2a

Eed

Kdm5b

Sall4

Terf2

Zbtb48

**0.0**

**2.7**

**5.5**

**8.7**

**10.0**

**0.0**

**1.0**

**3.4**

**7.8**

**8.6**

**0.0**

**3.4**

**3.7**

**4.5**

**5.5**

**0.0**

**5.0**

**4.6**

**5.6**

**6.1**

**0.0**

**0.8**

**0.6**

**4.8**

**5.5**

**0.0**

**-0.0**

**-0.0**

**3.6**

**4.6**

**0.0**

**-0.9**

**-0.6**

**3.4**

**3.4**

**0.0**

**0.3**

**-0.0**

**4.2**

**3.9**

**0.0**

**0.3**

**1.3**

**2.0**

**2.7**

**0.0**

**0.1**

**-0.7**

**1.5**

**1.9**

**0.0**

**0.7**

**0.3**

**1.7**

**2.1**

**0.0**

**-0.0**

**0.8**

**0.9**

**1.4**

**0.0**

**-0.1**

**0.1**

**0.5**

**0.8**

**0.0**

**0.3**

**-0.1**

**0.1**

**0.3**

**0.0**

**0.5**

**0.5**

**0.4**

**0.7**

**0.0**

**-0.1**

**0.0**

**0.3**

**0.3**

**0.0**

**0.1**

**-0.0**

**0.1**

**0.2**

**0.0**

**0.5**

**0.3**

**-0.3**

**0.2**

**0.0**

**-0.1**

**0.3**

**-0.1**

**0.6**

**0.0**

**-0.5**

**-0.3**

**0.2**

**0.5**

ESC

WT

c-JunKO

WT

c-JunKO

PNT-D3

EpiSC

EpiSC-like

**0**

**5**

**1**

**0**

**1**

**5**

DNA replication**^0.6^**

### 0.6

DNA repair**_1.3_** transcription, DNA-templated**^1.1^**

**^0.9^** histone H3 acetylation**_1.8_** regulation of stem cell population maintenance**2.0** inner cell mass cell proliferation**^1.2^**

**2.9**

**4.1**

-log10(P value) **^5.2^**

**5.4**

**7.2**

**8.1**

**7.7**

**6.6**

### 7.1

**10.0**

**10.0**

**S4. Knock out c-Jun activates pluripotent genes during primed to naïve transition.**

1. Venn diagrams showing the overlap genes between high expression genes in ESC and up-regulated genes in c-Jun knock out primed to naïve transition (PNT) day 3 cells. Fold Change > 1 was used to screen high expression genes between two samples.
2. Violin plots of the expression level for overlap genes (1688) in panel **A**. Data were converted to a Z score based on the row-wise SD for each gene.
3. Gene ontology analysis of the overlap genes (1688) in panel **A**. Analysis was performed using DAVID.
4. Heatmap showing the expression of 16 selected pluripotency genes from the overlap genes (1688) in panel **A**. RNA-seq data is shown as log2(Fold Change) relative to expression in EpiSC. The value of Log2(Fold Change) was shown in each block.

Figure S5 (Related to Figure 4)


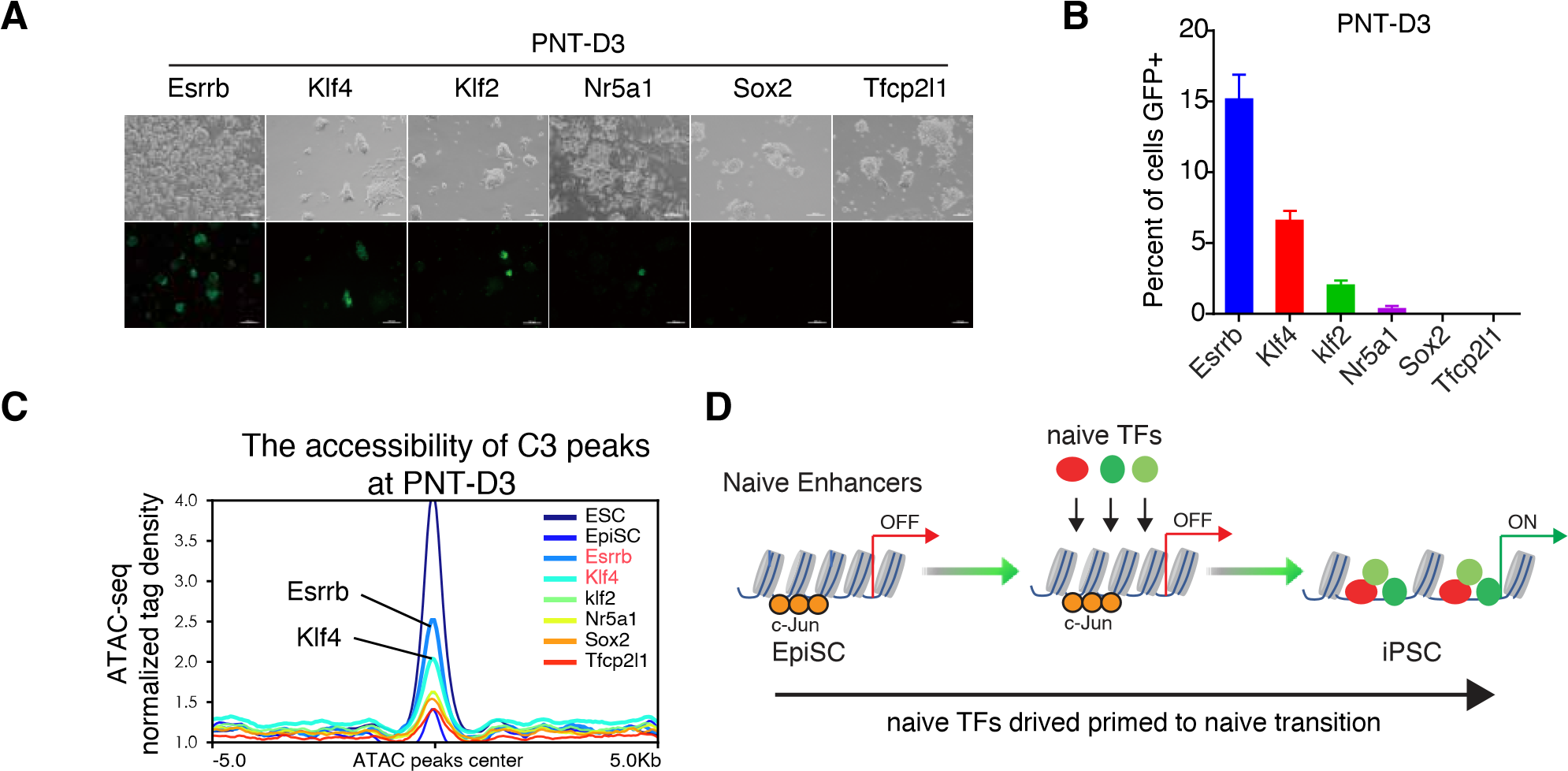


**S5. Esrrb promotes PNT by opening c-Jun locked naïve loci A.** Esrrb, Klf4, Klf2, Nr5a1, Sox2 and Tfcp2l1 were cloned into PiggyBac plasmid and each of the six PiggyBac plasmids was transfected into EpiSC and then perform PNT experiment. The number of iPSC colonies were count at PNTD3. D=day. Scale bar=100 μm.

1. Bar chart showing the percentage of cells GFP+ (GFP expressed from the

Oct4-GFP, OG2 reporter) during primed to naïve transition (PNT) by the defined TFs. Data are from 4 biological replicates in 2 independent experiments and are shown as the means ± SEM.

1. Average ATAC-seq read density at C3 peaks in ESC, EpiSC and cells of primed to naïve transition (PNT) induced by each of the six TFs at day 3 (PNTD3).
2. Working model of the naive TFs in reprogramming primed to naïve transition

(PNT). Naïve TFs were binding to the closed naïve enhancers which bind by cJun and opening the silenced naïve enhancers to activate the pluripotent program.
